# Inverse agonist of estrogen-related receptor α suppresses the growth of triple negative breast cancer cells through ROS generation and interaction with multiple cell signaling pathways

**DOI:** 10.18632/oncotarget.7276

**Published:** 2016-02-09

**Authors:** Ying-Min Wu, Zhuo-Jia Chen, Guan-Min Jiang, Kun-Shui Zhang, Qiao Liu, Shu-Wei Liang, Yan Zhou, Hong-Bin Huang, Jun Du, Hong-Sheng Wang

**Affiliations:** ^1^ Department of Microbial and Biochemical Pharmacy, School of Pharmaceutical Sciences, Sun Yat-Sen University, Guangzhou 510006, China; ^2^ Department of Pharmacy, Sun Yat-Sen University Cancer Center, State Key Laboratory of Oncology in South China, Collaborative Innovation Center for Cancer Medicine, Guangzhou 510060, China; ^3^ Hunan Cancer Hospital and The Affiliated Cancer Hospital of Xiangya School of Medicine, Central South University, Changsha 410013, China; ^4^ Department of Pharmacy, Sun Yat-Sen Memorial Hospital, Sun Yat-Sen University, Guangzhou 510120, China

**Keywords:** ERRα, XCT-790, TNBC, ROS, growth arrest

## Abstract

There is an urgent clinical need for targeted therapy approaches for triple-negative breast cancer (TNBC) patients. Increasing evidences suggested that the expression of estrogen-related receptor alpha (ERRα) was correlate with unfavorable clinical outcomes of breast cancer patients. We here show that inhibition of ERRα by its inverse agonist XCT-790 can suppress the proliferation, decrease G2/M phases, and induce mitochondrial-related apoptosis of TNBC cells. XCT-790 elevates the proteins related to endoplasmic reticulum (ER) stress such as ATF4/6, XBT-1 and CHOP. It also increases the expression of growth inhibition related proteins such as p53 and p21. Further, XCT-790 can increase the generation of reactive oxygen species (ROS) in TNBC cells mainly through inhibition of SOD1/2. While ROS scavenger NAC abolishes XCT-790 induced ER-stress and growth arrest. XCT-790 treatment can rapidly activate the signal molecules including ERK1/2, p38-MAPK, JNK, Akt, p65, and IκBα, while NAC attenuates effects of XCT-790 induced phosphorylation of ERK1/2, p38-MAPK and Akt. Further, the inhibitors of ERK1/2, JNK, Akt, and NF-κB attenuate XCT-790 induced ROS generation. These data suggest that AKT/ROS and ERK/ROS positive feedback loops, NF-κB/ROS, and ROS/p38-MAPK, are activated in XCT-790 treated TNBC cells. *In vivo* experiments show that XCT-790 significantly suppresses the growth of MDA-MB-231 xenograft tumors, which is associated with up regulation of p53, p21, ER-stress related proteins while down regulation of bcl-2. The present discovery makes XCT-790 a promising candidate drug and lays the foundation for future development of ERRα-based therapies for TNBC patients.

## INTRODUCTION

The estrogen-related receptor alpha (ERRα), a member of the nuclear hormone receptor super family of transcription factors, can regulate the energy metabolism and mitochondrial biogenesis [1]. Although the endogenous ligand has yet to be defined, ERRα can modulate the expression of transcriptomes of estrogen receptor (ER) due to the high degree of structural similarity in the DNA binding domain [2]. Given the established role of estrogen in breast cancer, the roles of ERRα in ER signaling and pathogenesis of breast cancer have been continuously concerned. Previous studies revealed that the expression of ERRα was correlate with increased risk of recurrence and adverse clinical outcomes of breast cancer patients via a ER status independent manner [3, 4]. The ChIP-chip analyses of breast cancer cells revealed that majority of the genes regulated by ERRα are distinct from those controlled by ER [5, 6]. Further, high ERRα expression has been reported in breast, ovarian and prostate cancers, which is correlated with poor prognosis [3, 7]. ERRα is also involved in angiogenesis and response to hypoxia of solid tumors [8, 9]. These rapidly accumulating evidences suggest that ERRα emerges as a transcriptional metabolic regulator that also promotes cancer development [10, 11].

Triple-negative breast cancers (TNBCs), which is clinically defined by the absence of ER, progesterone receptor (PR), or human epidermal growth factor receptor 2 (HER2), accounts for about 15∼20% of all newly diagnosed breast cancer cases [12]. TNBC is generally associated with high risk of disease recurrence and increased risk of visceral and cerebral metastases compared with other subtypes of breast cancer [13–15]. Further, patients are not eligible for endocrine or HER2 targeted therapies, thus rendering chemotherapy the only therapeutic option [16, 17]. The rate of death in patients with TNBC at 5 years is twice that of ERα positive tumors [18]. Considering there is no FDA (Food and Drug Administration) approved targeted therapy for TNBC patients, the investigation of underlying molecular mechanisms and exploitation of new targeted approaches are desperately needed.

ERRα is required for the growth and migration of breast cancer cells when assayed *in vitro* or propagated as xenografts [3, 7, 8, 19]. Our recent study revealed inhibition of ERRα can suppress the metastasis of TNBC cells via directly targeting fibronectin [20]. Further, ERRα expression indicates worse prognosis and correlated with poor outcome predictors in TNBC patients [21]. While the roles and mechanisms of ERRα in the progression and growth of TNBC remain unclear. XCT 790, which binds to the inferred ligand-binding domain of ERRα, is the selective inhibitor of ERRα. It has been widely used to investigate the biological effects of ERRα [20, 22, 23]. The primary objective of the present study is to illustrate the effects and related mechanisms of ERRα on the *in vitro* and *in vivo* growth of TNBC.

## RESULTS

### XCT-790 inhibits the proliferation and induces cell cycle arrest of TNBC cells

**Figure 1:**
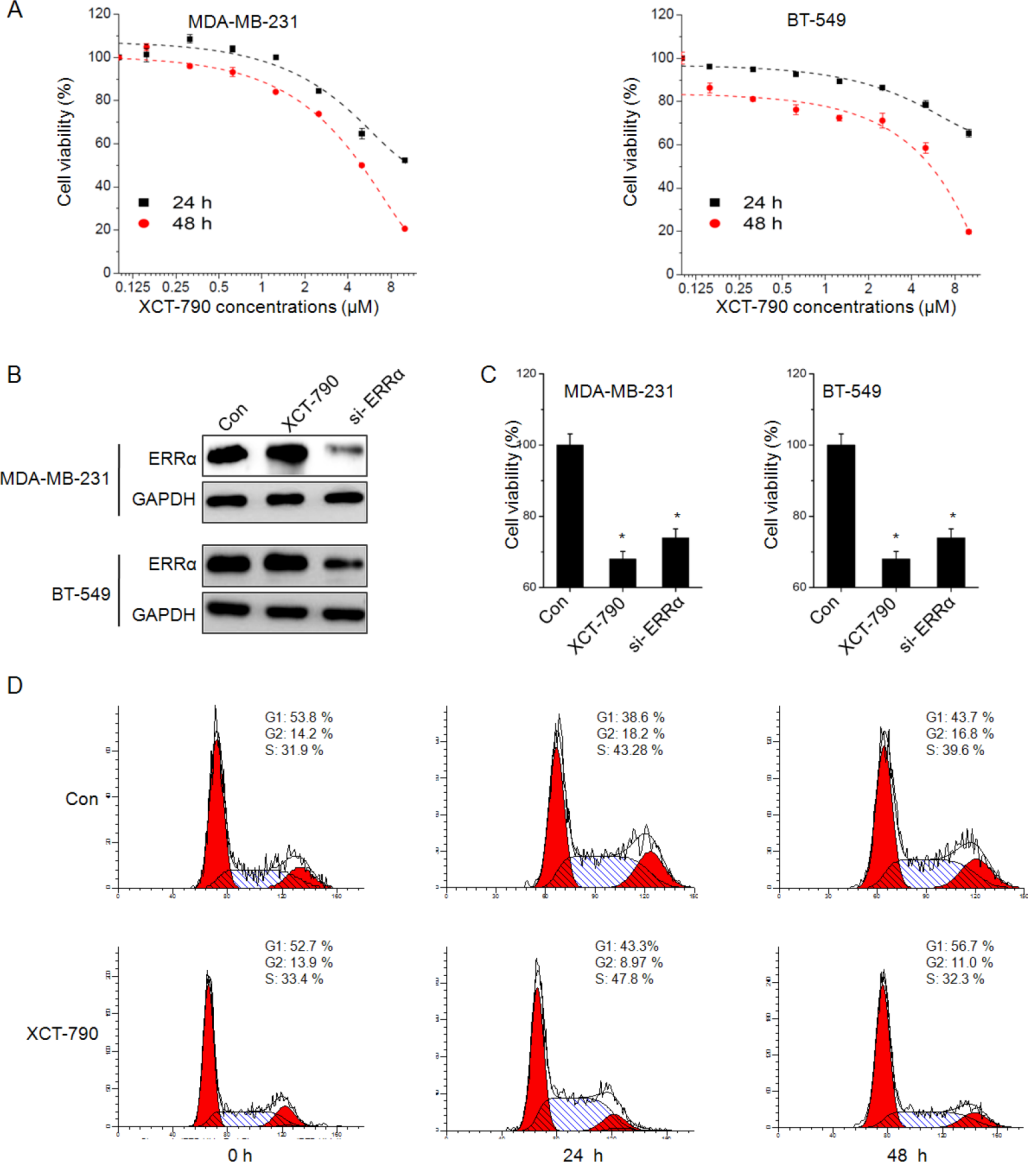
XCT-790 inhibits the proliferation and induces cell cycle arrest of TNBC cells (**A**) MDA-MB-231 or BT-549 cells were treated with various concentrations of XCT-790 for 24 or 48 h, and then cell viability was assessed by CCK-8 kit. After 24 h treatment with XCT790 (5 μM) or pre-transfection with si-NC or si-ERRα siRNAs, the protein levels of ERRα were analyzed by Western blot analysis (**B**), the cell viability of MDA-MB-231 or BT-549 cells was assessed by CCK-8 kit (**C**). (**D**) MDA-MB-231 cells were synchronized at the G1/S transition by a double TdR block, and then treated with 5 μM XCT-790 for the indicated times. The cycle cycles were analyzed by FCM. Data were presented as means ± SD of three independent experiments (Ten independent experiments for cell viability). *p < 0.05 compared with control group.

Our recent study revealed that ERRα was highly detected in TNBC MDA-MB-231 and BT-549 cells [20]. Then roles of ERRα inverse agonist XCT-790 on cell viability were further investigated. As shown in Figure 1A, XCT-790 treatment inhibited the proliferation of both MDA-MB-231 and BT-549 cells via a concentration-dependent manner. The IC_50_ values of XCT-790 (48 h) to MDA-MB-231 and BT-549 cells were 13.7 and 13.3 μM, respectively. Therefore 5 μM XCT-790 was chose for further studies on the basis of cytotoxicity test and other previous studies [5, 7]. To validate the essential roles of ERRα for XCT-790 induced suppression of TNBC cell proliferation, MDA-MB-231 and BT-549 cells were transfected with non-targeting control si-RNA or si-ERRα for 24 h. Western blot analysis revealed that the expression of ERRα was significantly silenced by si-ERRα while not XCT-790 (Figure 1B). The silencing of ERRα also suppressed the growth of both MDA-MB-231 and BT-549 cells (Figure 1C).

Whether XCT-790 blocked cells in a specific phase of cell cycle was further determined. We synchronized cells using double TdR-blocking method. Flow cytometry (FCM) analysis showed an obvious decrease in the percentage of cells in G2/M phase of XCT-790 treated MDA-MB-231 cells, as compared with that in DMSO (0.5%, v/v) treated control cells. The decrease of G2/M phases by XCT-790 lasted throughout 48 h treatment period (Figure 1D). Similar XCT-790 induced G2/M phase decrease was also observed in BT-549 cells (Data not shown). Collectively, these data revealed that inhibition of XCT-790 by XCT-790 can significantly inhibit the *in vitro* growth of TNBC cells by decreasing G2/M phases.

### XCT-790 induces mitochondrial-related apoptosis

**Figure 2:**
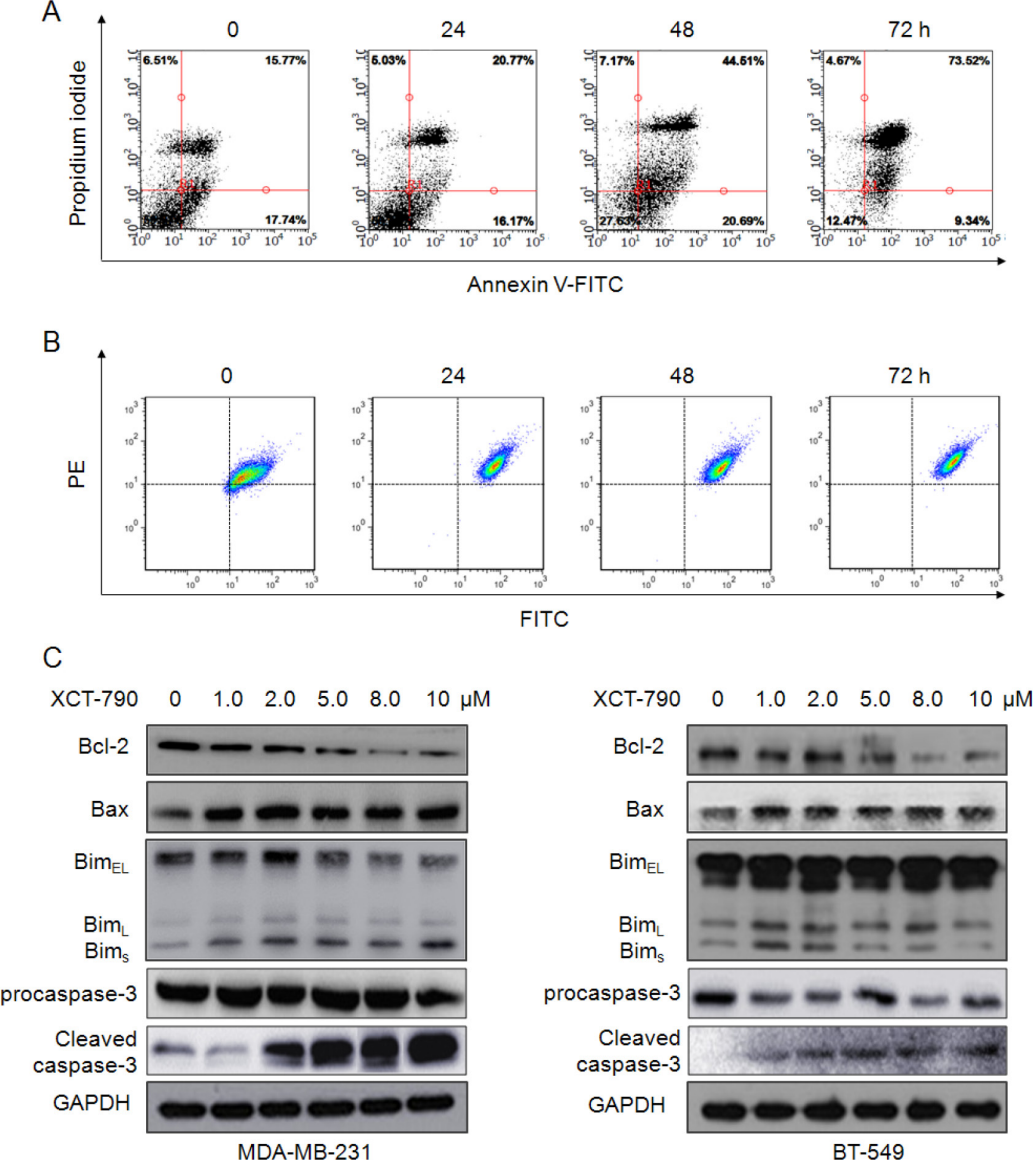
The inhibition of ERRα induces mitochondrial-related apoptosis in TNBC cells (**A**) MDA-MB-231 cells were treated with 10 μM XCT-790 for increased time periods, stained with annexin V-FITC and PI, and then analyzed by FCM for cell apoptosis. (**B**) MDA-MB-231 cells were treated with 10 μM XCT-790 as the indicated concentrations for 24 h, and then JC-1, the mitochondria-specific dye, was added to measure the membrane polarity (ΔΨm). Apoptotic cells mainly show green fluorescence (FITC), while healthy cells show red fluorescence (PE). (**C**) MDA-MB-231 and BT-549 cells were treated with XCT-790 as the indicated concentrations for 48 h, and then Bcl-2, Bax, and Bim protein expression levels were analyzed by Western blot analysis. Data were presented as means ± SD of three independent experiments.

Next, MDA-MB-231 cells were treated with 10 μM XCT-790 for increased time periods, and then apoptotic cells were detected by FCM. As shown in Figure 2A, XCT-790 treatment resulted in a marked time dependent increase in apoptosis of MDA-MB-231 cells. Further, the mitochondrial membrane potential (ΔΨ*m*) was measured using fluorochrome dye JC-1. Our results showed that XCT-790 treatment also resulted in a time dependent increase in the ratio of the green fluorescence to red fluorescence (Figure 2B), suggesting that inhibition of ERRα can decrease the ΔΨ*m* and thus promote cell apoptosis. The expression levels of apoptotic related proteins in TNBC cells were further measured. As shown in Figure 2C, inhibition of ERRα significantly (*p* < 0.05) up regulated the expression of Bax, Bim and cleaved caspase-3 while down regulated the expression of Bcl-2 and procaspase-3 in both MDA-MB-231 and BT-549 cells, which led to an decrease of antiapoptotic/proapoptotic Bcl-2/Bax ratios (Figure S1). Collectively, these data suggested that the mitochondrial-related apoptosis is involved in XCT-790 induced TNBC cell growth arrest.

### XCT-790 increases ROS generation by suppressing SOD1/2

**Figure 3:**
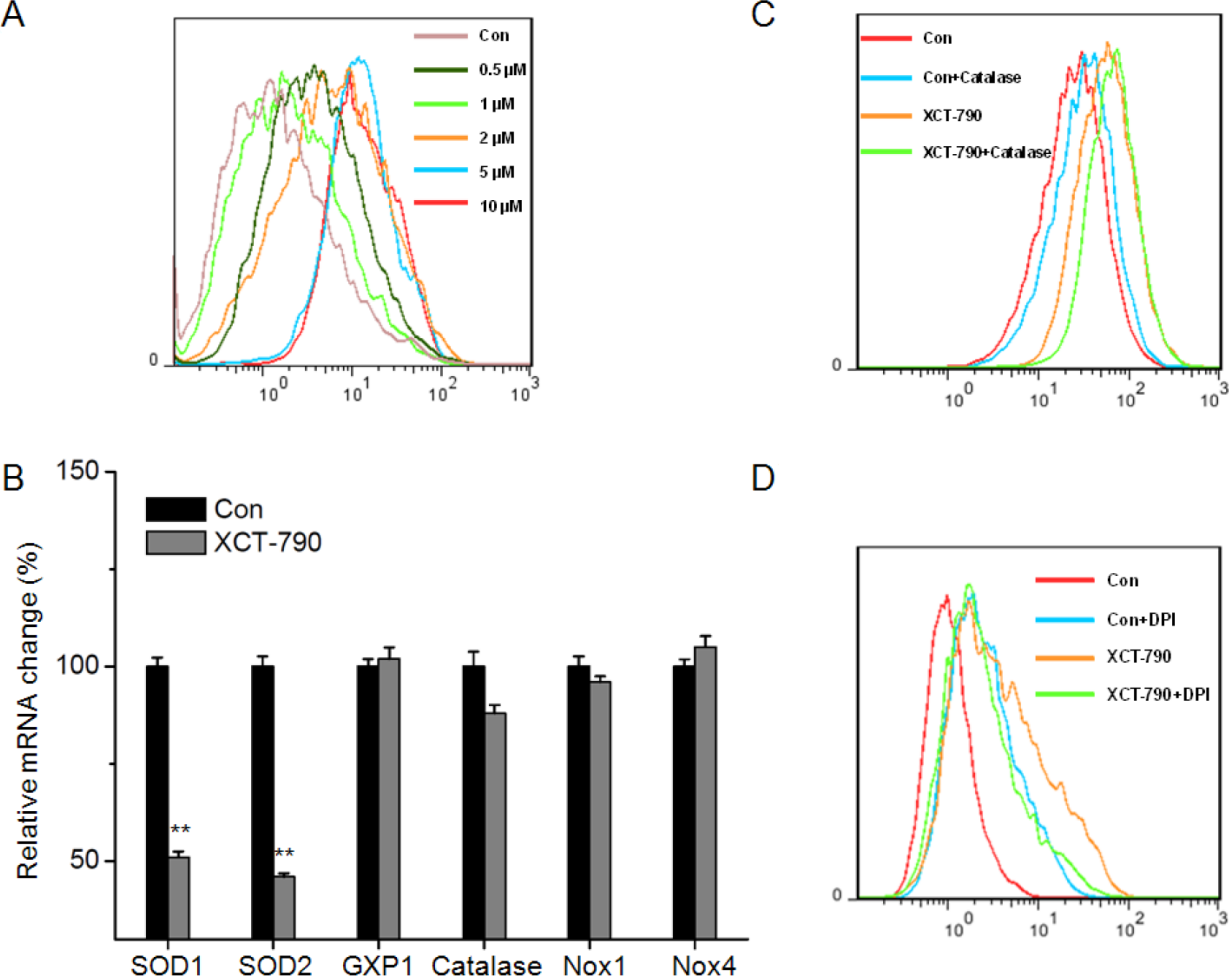
XCT-790 increases ROS generation by suppressing SOD1/2 (**A**) MDA-MB-231 cells were treated with increasing concentrations of XCT-790 for 12 h, and then loaded with CM-H2DCFDA. The fluorescence intensity was measured by FCM. (**B**) MDA-MB-231 cells were treated with 5 μM XCT-790 for 12 h, and then the mRNA levels of SOD1, SOD2, GXP1, Catalase, NOX1, and NOX4 were measured by qRT-PCR; MDA-MB-231 cells were pretreated with or without 2000 U Catalase (**C**) or 10 μM DPI (**D**) for 1 h, and then exposed to XCT-790 for 12 h. Data were presented as means ± SD of three independent experiments.

ROS generation plays an important role in apoptotic effects of anticancer agents [24, 25]. To determine whether ROS generation is involved in XCT-790-induced apoptosis, we measured the levels of ROS in XCT-790 treated cells by use of DCF-DA. Our results revealed that XCT-790 can increase the ROS generation via a does dependent manner in MDA-MB-231 cells (Figure 3A). We analyzed the expression of relevant antioxidant enzymes such as SOD, GXP1 or catalase and ROS generation enzymes including NOX1 and NOX4 [26]. As shown in Figure 3B, XCT-790 obviously decreased the mRNA expression of SOD1 and SOD2 while not GXP1, catalase, NOX1, or NOX4. This was confirmed by the results that catalase (H_2_O_2_ scavenger) (Figure 3C) or diphenyleneiodonium chloride (DPI, NOX inhibitor) (Figure 3D) had no significant effect on XCT-790 induced ROS generation of MDA-MB-231 cells. Therefore, our results revealed that XCT-790 increases ROS generation mainly by suppressing SOD1/2 expression.

### Endoplasmic reticulum (ER) stress is involved in XCT-790 induced growth arrest

**Figure 4:**
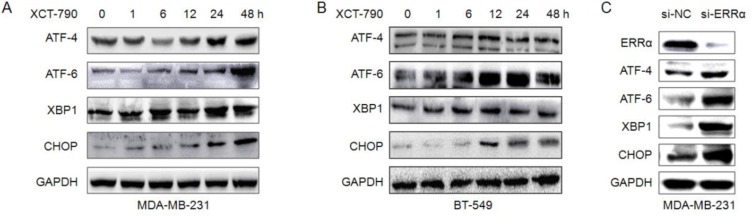
ER stress is involved in XCT-790 induced apoptosis MDA-MB-231 (**A**) or BT-549 (**B**) cells were treated with 5 μM XCT-790 for the indicated times, and then the expression of ATF4, ATF6, XBP-1 and CHOP were determined by Western blot analysis; (**C**) MDA-MB-231 cells were transfected with siNC or si-ERRα for 48 h, and then the ER-stress related proteins were measured by Western blot analysis.

In addition to mitochondrial pathway, ER stress also plays an important role in cancer cell growth arrest and apoptosis [27, 28]. We examined the expressions of ER stress-related proteins such as activating transcription factor 4 (ATF4), activating transcription factor 6 (ATF6), X-box binding proteins 1 (XBP-1), and C/EBP-homologous protein (CHOP) in XCT-790-treated TNBC cells. The results indicated that the protein levels of ATF4, ATF6, XBP-1, and CHOP were obviously increased after treatment for 12 h. The up regulation of these proteins can last more than 48 h in both MDA-MB-231 (Figure 4A) and BT-549 (Figure 4B) cells. Furthermore, si-ERRα also obviously increased the expression of ATF4, ATF6, XBP-1, and CHOP in MDA-MB-231 cells (Figure 4C). These data suggested that ER stress is involved in XCT-790 induced growth arrest of TNBC cells.

### XCT-790 increases the expression of growth inhibition related proteins

**Figure 5:**
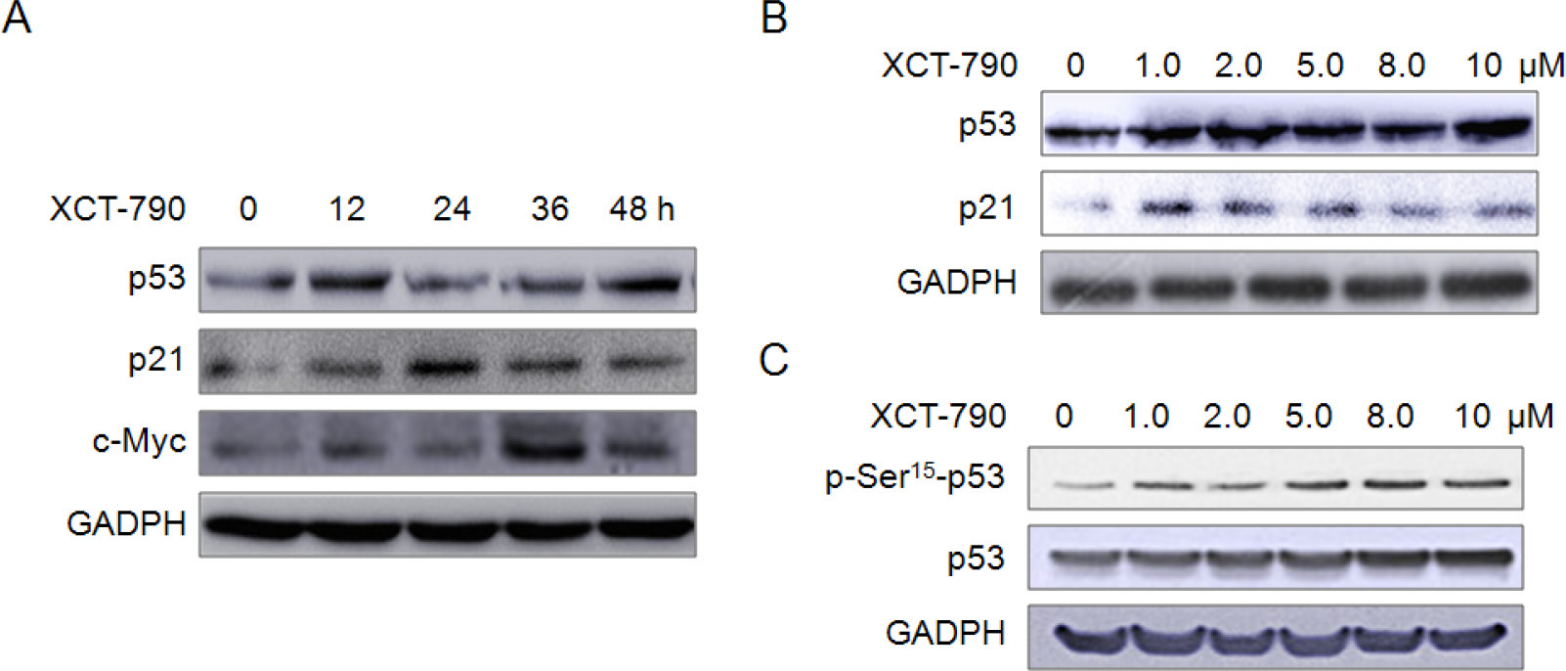
XCT-790 increases the expression of growth inhibition related proteins (**A**) MDA-MB-231 cells were treated with 5 μM XCT-790 for the indicated times, and then the expression of p53, p21 and c-Myc were measured by Western blot analysis; (**B**) MDA-MB-231 cells were treated with increasing concentrations of XCT-790 for 24 h, then the expression of p53 and p21 were measured by Western blot analysis; (**C**) MDA-MB-231 cells were treated with increasing concentrations of XCT-790 for 1 h, the total and phosphorylation of p53 were measured by Western blot analysis.

Proteins such as p53, p21 and c-Myc have been implicated in apoptosis and mitochondrial dysfunction. We thus examined the effects of XCT-790 on the expression of these proteins. We found that XCT-790 can increase the protein levels of p53 and p21 via both time (Figure 5A) and concentration (Figure 5B) dependent manners. Further, XCT-790 treatment also significantly (*p* < 0.05) increased the levels of p-Ser^15^-p53 via a concentration dependent manner (Figure 5C), which is essential for translocation of p53 to the nucleus [29]. These results clearly demonstrated that XCT-790 can impair growth and induce apoptosis via up-regulation of apoptotic proteins in TNBC cells.

### ROS is involved in XCT-790 induced ER stress and growth arrest

**Figure 6:**
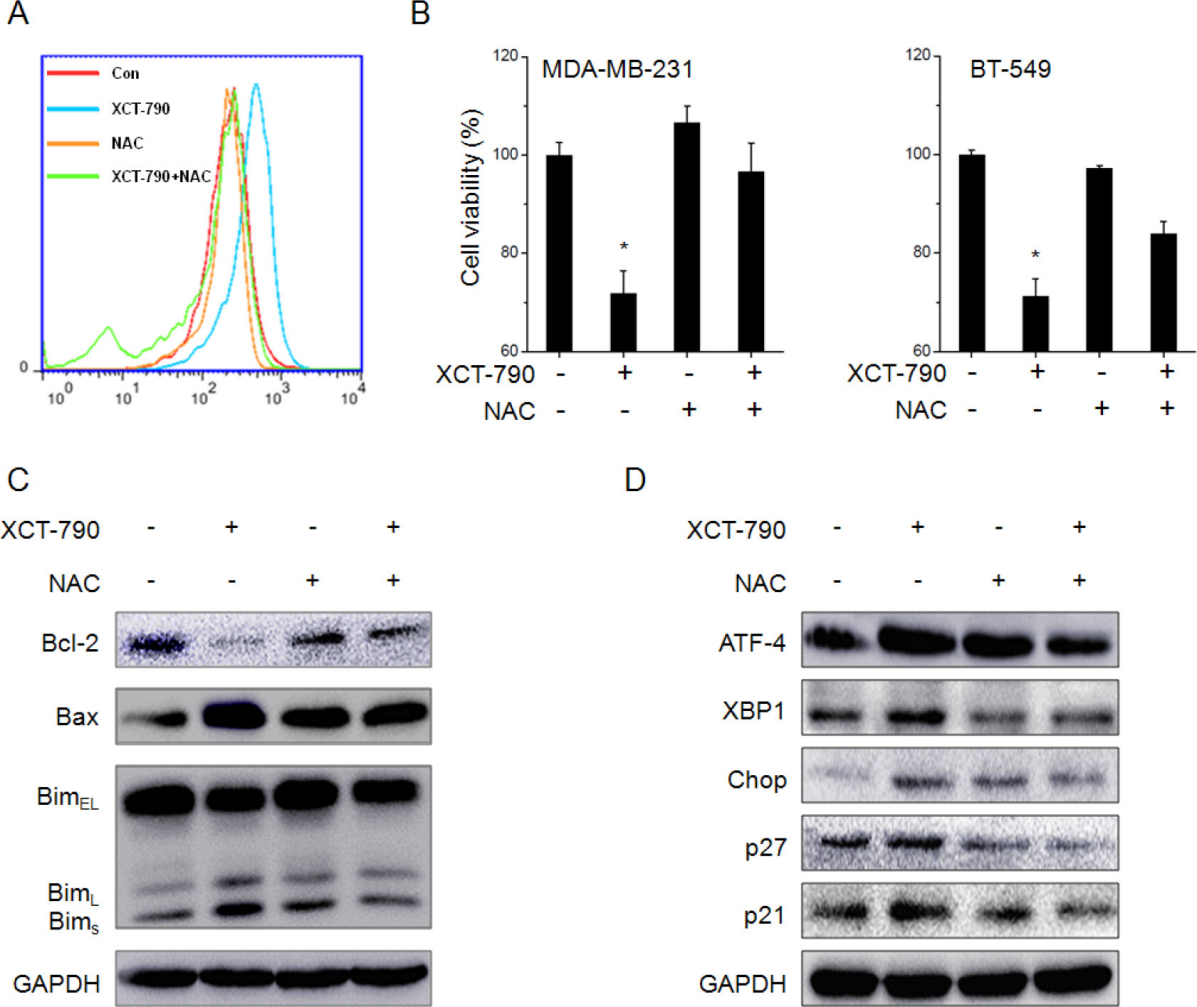
ROS mediates XCT-790 induced ER stress and growth arrest (**A**) MDA-MB-231 cells were pretreated with NAC (20 mM) for 1 h and then treated with 5 μM XCT-790 for 12 h, the ROS was measured by FCM; (**B**) MDA-MB-231 or BT-549 cells were pretreated with NAC (20 mM) for 1 h and then treated with 5 μM XCT-790 for 48 h, the cell viability was measured by use of CCK-8 kit; (**C & D**) MDA-MB-231 cells were pretreated with NAC (20 mM) for 1 h and then treated with 5 μM XCT-790 for 24 h, the protein expression was examined by Western blot analysis. Data were presented as means ± SD of three independent experiments. *p < 0.05 compared with control group.

We then assessed the roles of ROS generation in XCT-790 induced ER stress and growth arrest. Our results revealed that ROS scavenger NAC markedly attenuated XCT-790 induced ROS generation in MDA-MB-231 cells (Figure 6A). NAC also alleviated the inhibition effects of XCT-790 on the proliferation effects of MDA-MB-231 and BT-549 cells (Figure 6B). We also examined the effects of NAC on XCT-790 induced variation of growth arrest, apoptosis, and ER stress related proteins. Our results revealed that NAC obviously attenuated XCT-790 induced down regulation of Bcl-2 and up regulation of Bax, Bims, ATF-4, XBP-1, CHOP, p21 and p27 (Figure 6C and 6D). The results suggested that ROS is involved in XCT-790 induced ER stress and growth arrest.

### XCT-790 activates MAPK, PI3K/Akt, NF-κB signals via ROS dependent or independent manner

**Figure 7:**
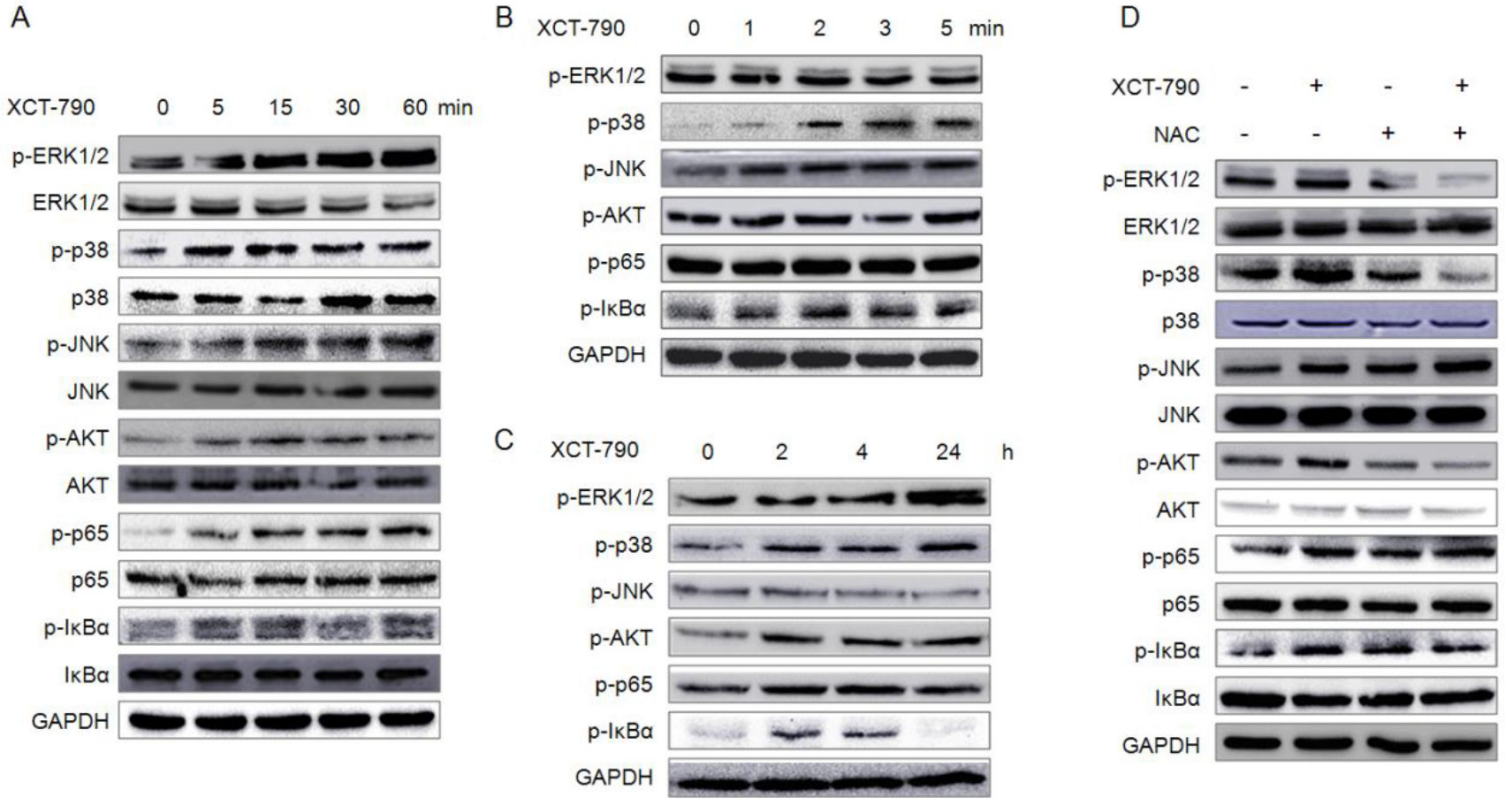
XCT-790 activates MAPK, PI3K/Akt, NF-κB signals via ROS dependent or independent manner (**A**–**C**) MDA-MB-231 cells were treated with 5 μM XCT-790 for the indicated times, and then the phosphorylation and total expression of these signal molecules were measured by Western blot analysis; (**D**) MDA-MB-231 cells were pretreated with NAC (20 mM) for 1 h and then treated with XCT-790 for 30 min, the phosphorylation and total expression of MAPK, Akt, NF-κB were measured by Western blot analysis.

We next sought to identify the signal transduction pathways involved in proliferation inhibition in response to XCT-790 treatment. We monitored the expression levels and activation statuses of MAPKs, PI3K/Akt, and NF-κB, which have been implicated in cell proliferation and apoptosis [30, 31]. As shown in Figure 7A–7C, XCT-790 treatment rapidly activated (1 min) JNK and lasted for 60 min. For I*κBα*, XCT-790 activated it since 1 min and lasted for 4 h. As to p38-MAPK, XCT-790 activated it at 2 min and lasted for more than 24 h. As to ERK1/2, Akt, and p65, XCT-790 activated them since 5 min and also lasted for more than 24 h. While XCT-790 treatment had no obvious effect on the total levels of ERK1/2, p38-MAPK, JNK, Akt, p65 or I*κBα* for 0 to 24 h (Figure S2). In addition, our results confirmed that XCT-790 treatment increased the transcription activities of NF-κB in MDA-MB-231 cells by use of dual luciferase reporter assay (Figure S3). Furthermore, the ROS scavenger NAC significantly attenuated XCT-790 induced activation of ERK1/2, p-38 MAPK, and Akt, while not JNK, p65, or I*κBα. It suggested that XCT-790 can activate the ERK1/2*, p-38 MAPK and Akt via ROS dependently and JNK, NF-κB via ROS independently.

### MAPK and NF-κB signals participate XCT-790 induced ROS generation and growth arrest

**Figure 8:**
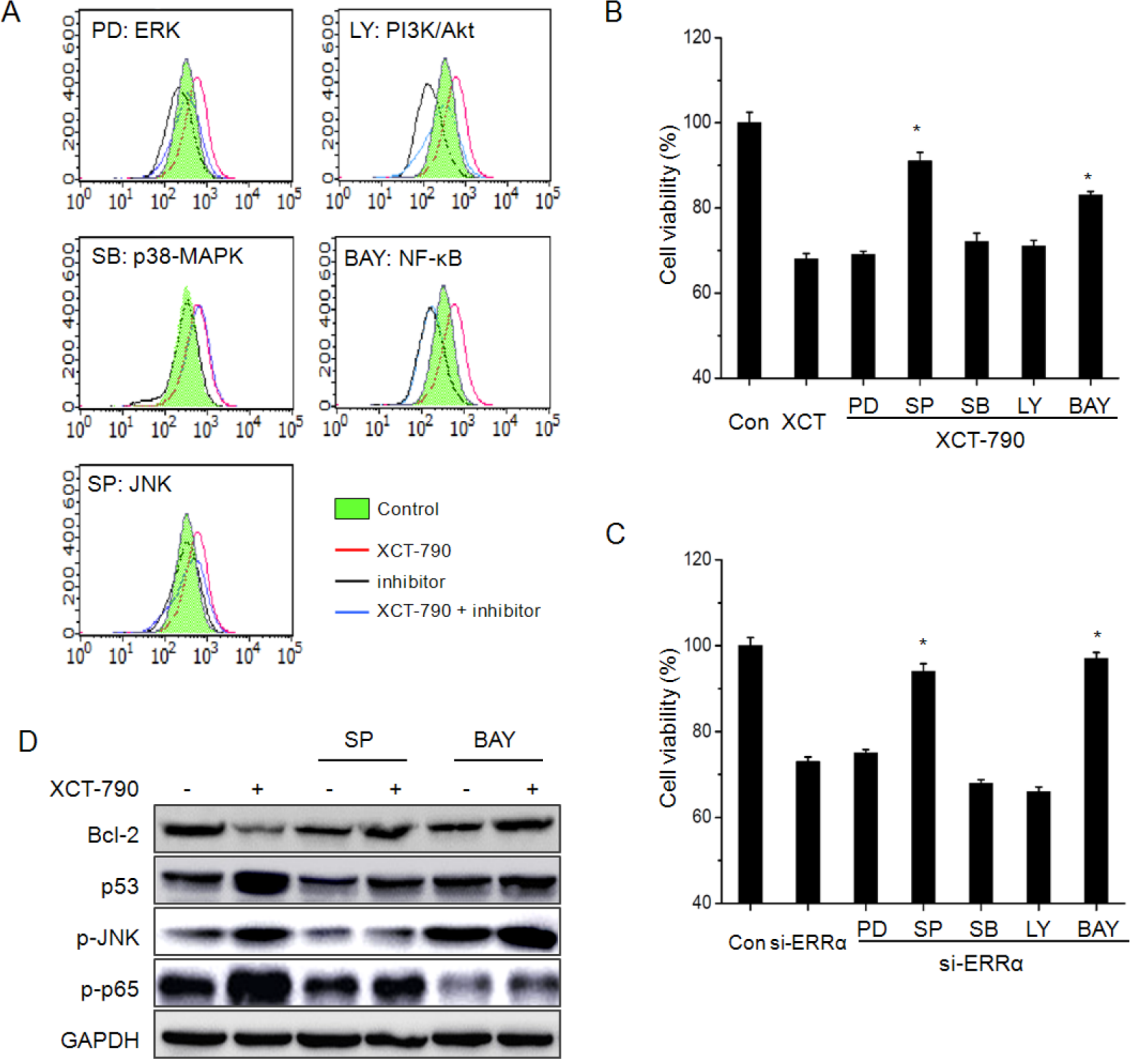
MAPK and NF-κB signals participate XCT-790 induced ROS generation and growth arrest MDA-MB-231 cells were pretreated with 10 μM ERK1/2 inhibitor PD98059 (PD), JNK inhibitor SP600125 (SP), p38-MAPK inhibitor SB203580 (SB), PI3K inhibitor LY294002 (LY), or NF-κB inhibitor BAY11-7082 (BAY) for 90 min, and then exposed to 5 μM XCT-790 for further 12 h (**A**) or 48 h (**B**), the ROS and cell viability were measured by FCM and CCK-8 assay, respectively; (**C**) MDA-MB-231 cells were pretreated with inhibitors for 90 min, and then transfected with si-ERRα for another 48 h, the cell viability was measured by CCK-8 assay; (**D**) MDA-MB-231 cells were pretreated with SP or BAY for 90 min and then stimulated with XCT-790 for further 24 h, the expression of Bcl-2, p53, p-JNK, and p-p65 were measured by Western blot analysis. Data were presented as means ± SD of three independent experiments. *p < 0.05 compared with XCT-790 or si-ERRα group.

We next investigated whether MAPK, PI3K/Akt, NF-κB signals were involved in XCT-790 induced ROS generation and growth arrest of TNBC cells. The results revealed that the all inhibitors excluding SB (p-38 MAPK inhibitor) significantly attenuated XCT-790 induced ROS generation (Figure 8A). Further, cell viability assays showed that the inhibitor of JNK and NF-κB, while not others, significantly alleviated the inhibition effects of XCT-790 on the proliferation of MDA-MB-231 cells (Figure 8B), while the inhibitor alone had no significant effect on cell proliferation (Figure S4). This was further confirmed by the results that SP and BAY attenuated the suppression effects of si-ERR*α* on the proliferation of MDA-MB-231 cells (Figure 8C). Further, the inhibitor of JNK and NF-κB also attenuated the down regulation of Bcl-2 and up regulation of p53 in MDA-MB-231 cells (Figure 8D). The results of cell viability revealed that p38-MAPK and NF-κB are involved in XCT-790 induced ROS generation and cell growth arrest, furthermore, ERK1/2 and PI3K/Akt also participate in XCT-790 induced ROS generation.

### XCT-790 inhibits TNBC xenograft tumor growth *in vivo*

**Figure 9:**
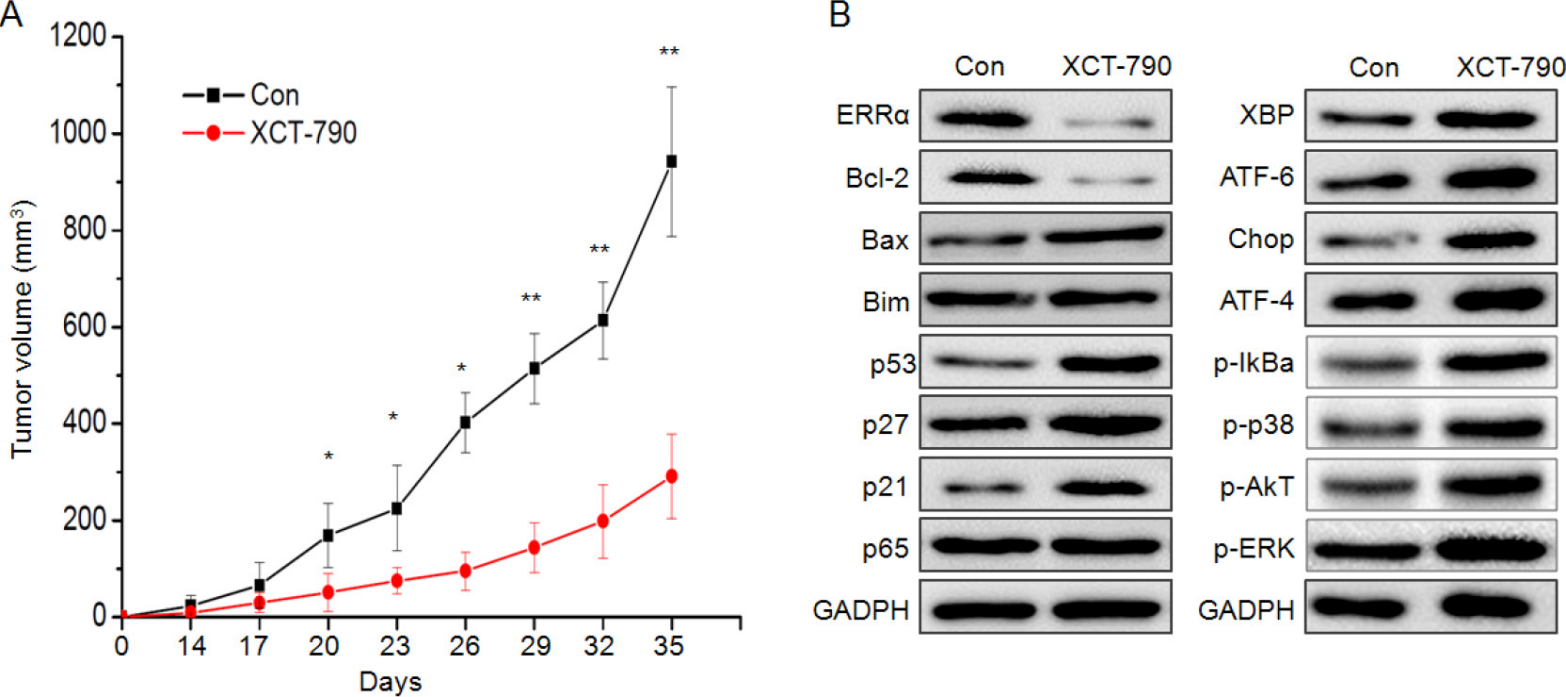
Inhibition of ERRα by XCT-790 inhibits the MDA-MB-231 xenograft growth *in vivo* (**A**) Tumor size was measured at the indicated time intervals. (**B**) The proteins related to the growth inhibition effects of XCT-790 were determined by Western blot analysis in the tumor lysates from the control and XCT-790 treated group. *p < 0.05 compared with control; **p < 0.01 compared with control.

We examined the effect of XCT-790 on the progression of MDA-MB-231 tumor xenografts in nude mice. Cells began to form measurable tumors after 14 days. After 35 days, all mice were sacrificed due to the large volume of tumor in the control group. During all the processes of experiments, the average size of tumor in XCT-790 groups was significantly (*p* < 0.05) less than that of control ones (Figure 9A). At the end of the experiment, the volume of control group (941 ± 154 mm^3^) was significantly greater than that in XCT-790 group (292 ± 87.1 mm^3^). This indicated that XCT-790 is able to inhibit the *in vivo* growth of TNBC cells. Western blot analysis showed that XCT-790 treatment significantly enhanced the expression of apoptosis related proteins p-p53, p53, p27, p21, ER stress related proteins XBP, ATF-4/6, XBP, CHOP, and activated the signal molecules I*κBα, p38-MAPK, Akt, and ERK*, while decreased the expression of ERRα and Bcl-2 (Figure 9C). These data suggested that XCT-790 can inhibit the growth of TNBC in nude mice bearing MDA-MB-231 xenografts via proliferation suppression and apoptosis induction.

## DISCUSSION

ERRα has been suggested to be a key factor for energy metabolism and progression of various cancers [3, 7]. Herein, we provide new insights into the protumorigenic action of ERRα on the progression of TNBC, an aggressive and deadly type of cancer threatening patients all over the world. Using the specific inverse agonist of ERRα, we demonstrate that XCT-790 suppresses the proliferation, decreases G2/M phases, and induces mitochondrial-related apoptosis of TNBC cells. Further, XCT-790 also increases the expression of ER-stress and growth inhibition related proteins and induces the ROS generation mainly through suppression of SOD1/2. Our data also show that the ROS generation mediates XCT-790 induced ER-stress and growth arrest. Multiple signal pathways, such as AKT/ROS and ERK/ROS positive feedback loops, NF-κB/ROS, and ROS/p38-MAPK, participate XCT-790 induced growth arrest. In MDA-MB-231 tumor xenografts in nude mice, XCT-790 treatment can significantly delay the *in vivo* growth of TNBC cells.

ERRα has been suggested to be correlated with unfavorable outcome in various cancer types such as prostate, colorectal, cervical and ovarian carcinomas [10, 11]. As to the breast cancer, high ERRα expression has been found to correlate with poor clinical outcome and/or unfavorable biomarkers such as ErbB2 [4, 5]. While ERRα deficiency significantly delays tumor formation in a mouse model of ErbB2-induced mammary tumorigenesis [32]. Our study reveals that inhibition of ERRα by its specific inverse agonist XCT-790 significantly inhibits the *in vitro* and *in vivo* growth of TNBC cells. It is consistent with previous study that inhibition of ERRα by its specific siRNA or XCT-790 can suppress the growth of colon [33], prostate [7], and estrogen receptor positive or negative breast [19, 34] cancer cells. Furthermore, our previous and others’ studies indicated the inhibition of ERRα can also suppress the migration and invasion of cancer cells via suppression the epithelial-to-mesenchymal transition [20, 35], down regulation the stability of RHOA [36] or induction the expression of WNT11 [37]. Collectively, these data suggested that ERRα plays a positive role in the progression of TNBC, while its inhibition might be a potential valuable approach for TNBC treatment.

Our study suggests that ROS serves as a critical role in XCT-790 induced growth arrest and ER stress of TNBC cells. *First*, XCT-790 induces ROS production in TNBC cells via a concentration dependent manner. *Second*, ROS scavenger is able to suppress ROS production. *Third*, ROS scavenger can attenuate XCT-790 induced down regulation of Bcl-2 and up regulation of ER stress and apoptotic related proteins. *Fourth*, ROS scavenger abolishes XCT-790 induced growth arrest *in vitro*. The elevation of ROS can be utilized to suppress cancer cell population growth and even induce apoptosis [24, 25]. Previous studies revealed that ERRα is essential for mitochondrial function and the expression of antioxidant protection genes [38]. XCT-790 can arrest A549 lung cancer cell population growth by inducing mitochondrial ROS production [39]. Considering that cancer cells are more sensitive to rapid increases of ROS levels than normal cells [40], our study provides a plausible mechanistic explanation for that targeted inhibition of ERRα to suppress the progression of TNBC via a ROS-dependent manner, which will selectively kill cancer cells without causing significant toxicity to normal cells [41]. Further, our results, together with other previous studies, suggested that ERRα inverse agonist would potentially have therapeutic value as novel chemotherapeutics for treating TNBC [19, 20, 39].

The role of ER stress in ROS-induced apoptosis has been demonstrated in a variety of cell types [27, 28]. As the results of oxidative stress mediated by ROS, accumulation of unfolded or misfolded proteins triggers a cellular adaptive procedure known as ER stress. Among the ER-stress related proteins, ATF4, ATF6, XBP-1, and CHOP are typical ER stress-regulated proteins involved in ER stress-induced apoptosis [42, 43]. Our result reveal that XCT-790 can up regulate the expression of ATF4, ATF6, XBP-1, and CHOP for more than 48 h in TNBC cells. The persistent or excessive ER stress inducing irreversible damages to cells is favorable to the activation of apoptosis [44]. Further, the ROS scavenger significantly attenuates XCT-790 induced ER-stress of TNBC cells, suggesting that ROS acts as upstream signals to initiate XCT-790-induced ER stress [45]. Collectively, our data reveal that XCT-790 induced ER stress is another pathway responsible for its inhibition effects on TNBC progression.

We then try to probe the mechanism for ROS generation and ant-cancer effects of XCT-790. We find that XCT-790 can significantly suppress the mRNA expression of SOD1/2, while not GXP1, Catalase, or NOX1/4. In the present study, neither DPI (NOX inhibitor) nor Catalase treatment can suppress XCT-790 induced ROS production, suggesting that NOX or Catalase might not be involved in XCT-790 induced ROS generation. As an important antioxidant enzyme, SOD1/2 plays a critical role in the reduction of intracellular oxidative stress [46]. Therefore, our study suggests that XCT-790 induced ROS generation mainly through suppression of SOD1/2.

Multiple signaling molecules, such as MAPKs, PI3K/Akt, and NF-*κB*, have been shown to regulate the generation of ROS [47]. Further, ROS functions as second messengers in diverse signaling pathways [48]. The MAPKs, including ERK1/2, JNK and p38 MAPK, are a family of serine/threonine kinases that regulate a variety of cellular events such as proliferation and apoptosis [30, 31]. It is well known that activation of JNK contributes to stress-induced apoptosis [49]. Here we find that XCT-790 can activate ERK1/2, p38α, and JNK1/2, but activation of p38α was not responsible for XCT-790 induced ROS generation. This is strongly supported by the findings that inhibition of JNK with SP600125 or ERK1/2 with PD98059 abolished XCT-790 induced ROS generation. Further, our study shows that pre-treatment with NAC prevented XCT-790 induced phosphorylation of ERK1/2 and p38-MAPK, while not JNK, indicating that ROS/ERK1/2 positive feedback, JNK/ROS, and ROS/p38-MAPK are involved in XCT-790 induced growth arrest of TNBC cells. Our previous study also revealed that sustained activation of ERK1/2 can suppress the proliferation and *in vivo* growth of breast cancer cells [50]. Further research is needed to address whether ERK and JNK induces ROS generation is responsible for the activation of p38-MAPK.

Our study reveals that ROS/Akt feedback loop and NF-κB/ROS pathway are also involved in XCT-790 induced ROS generation. Mounting evidences suggest that PI3K/Akt mediates the increase of ROS [51]. ROS scavenger NAC is able to suppress XCT-790 induced activation of Akt in MDA-MB-231 cells. Further, results show that XCT-790 rapidly (less than 1 min) induced IκBα phosphorylation, which is concomitant with IκB degradation, p65 phosphorylation and p65 nuclear translocation. Inhibition the activities of NF-κB and Akt significantly abolish XCT-790 induced ROS generation. Previous findings suggested dual roles for NF-κB signal transduction pathway in the regulation of cell growth. Paradoxically NF-κB has been known to be activated by various chemotherapeutic agents that induce growth arrest [52, 53], which is consistent with our present study. The activation of ROS/Akt signaling cascade is also required for chemotherapeutic agents induced growth and apoptosis [54]. Activated AKT serves as an upstream signaling molecule for NF-κB through IκBα kinase (IKK) and IκBα [55]. The relationship and cross talks between NF-κB and PI3K/Akt in XCT-790 induced ROS generation need further study.

**Figure 10:**
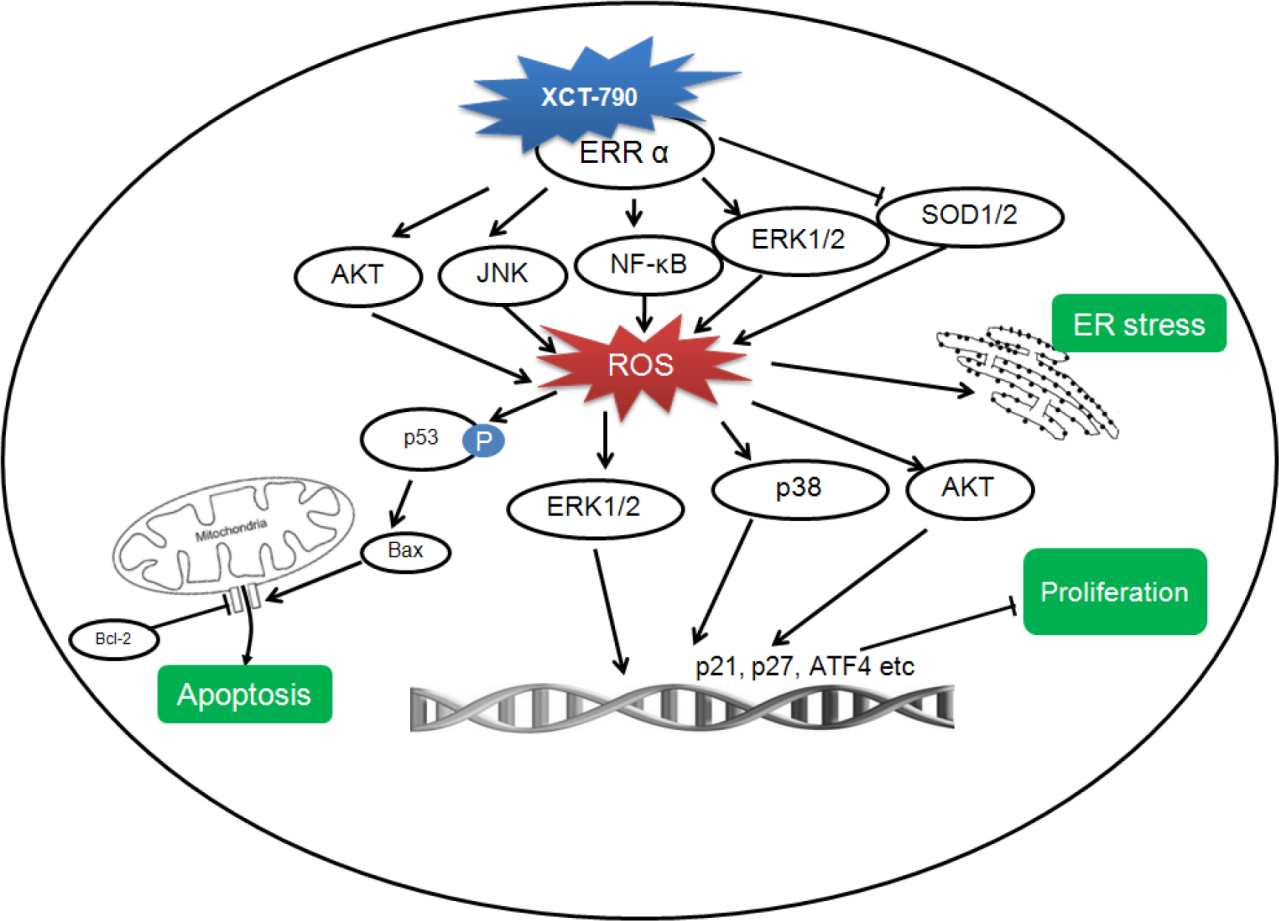
A proposed model to illustrate the mechanism of XCT-790 mediated growth arrest and apoptosis of TNBC cells

In summary, the ensemble of evidence presented in the current study demonstrates that the inverse agonist of ERRα can suppress the *in vitro* and *in vivo* growth of TNBC cells via elevation of ROS generation as summarized in Figure 10. Further, XCT-790 can induce mitochondrial-related apoptosis, increase the expression of various proteins involved in growth inhibition, and cause ER-stress of TNBC cells. Multiple signal pathways including AKT/ROS and ERK/ROS positive feedback loops, NF-κB/ROS, and ROS/p38-MAPK, participate the inhibition effects of XCT-790 on TNBC progression. However, the relationship and cross talks between these signal pathways warrant further study. Considering that there is no efficiency therapy target for TNBC until now, the present study not only strongly suggests that ERRα can be considered as a potential important target but also provides XCT-790 as a drug candidate for TNBC therapy.

## MATERIALS AND METHODS

### Reagents

PD 98059 (PD, ERK1/2 inhibitor), SP600125 (SP, JNK inhibitor), SB203580 (SB, p38-MAPK inhibitor), LY294002 (LY, PI3K/Akt inhibitor), BAY11-7082 (BAY, NF-κB inhibitor) were obtained from Sigma-Aldrich (St Louis, Mo., USA). XCT-790 (specific inverse agonist of ERRα) and other chemicals were of reagent grade or better and purchased from Sigma Chemical Co. (St. Louis, MO, USA) unless otherwise noted. Monoclonal antibodies were purchased from Cell Signaling Technology Inc. (Beverly, MA, USA) except the p-Akt and Akt, which were purchased from Bioworld Technology Inc (Minneapolis, MN, USA). Horseradish peroxidase-conjugated secondary antibody was obtained from Santa Cruz Biotechnology (Santa Cruz, CA, USA). All compounds were solubilized in dimethyl sulfoxide (DMSO). Steroid-free medium containing DMSO (0.5% v/v) was used as the control.

### Cell lines and culture

TNBC cell MDA-MB-231 and BT-549 were purchased from the American Type Culture Collection (Manassas, VA, USA) and cultured in RPMI medium 1640 (Invitrogen Corporation, Carlsbad, CA, USA) supplemented with 10% heat-inactivated fetal Bovin serum, 100 U/ml penicillin, and 10 μg/ml streptomycin at 37°C in a 5% CO_2_ atmosphere. An ABI 3130 Genetic Analyzer (Applied Biosystems) was used for the profiling. The DNA profile data were cross-checked with the ATCC data bank. Medium was replaced with phenol red-free medium 24 h before experiments to remove the estrogen-like activity of phenol red.

### Cell viability assay

Proliferation and viability of cells were detected by use of previously described procedures [56, 57]. Briefly, cell viability was evaluated by use of the CCK-8 kit (Dojindo Molecular Technologies, Gaithers burg, MD, USA) according to the manufacturer's instructions. The 50% inhibitory concentration (IC_50_) was calculated using GraphPad Prism software (GraphPad Software Inc., La Jolla, CA).

### Analysis of cell cycle, apoptosis, mitochondrial membrane potential (ΔΨm), and reactive oxygen species (ROS)

The assays of cell cycle, apoptosis, ΔΨm, and ROS were conducted as our previous methods [50]. Briefly, for cell cycle analysis, cells were synchronized at the G1/S transition by a double TdR block, as follows: 16 h block with 2.5 μM TdR (Sigma), 10 h release followed by the second block for 16 h. Then cells were treated with or without XCT-790, washed with PBS, fixed with 70% ethanol overnight at 4°C, incubated with propidium iodide (50 μg/mL), and analyzed by a Coulter Epics XL Flow Cytometry System (Beckman-Coulter, Miami, USA). For cell apoptosis analysis, both the suspension and the adherent cells were collected after XCT-790 treatment, stained with Annexin V-FITC for 15 min and propidium iodide (PI) for 5 min, and analyzed immediately by flow cytometry using FL1 (Em: 525 nm) and FL3 (Em: 670 nm). For ΔΨm measurement, JC-1 staining solution (5 μg/ml) was added to XCT-790 treated cells at 37°C for 20 min. After washed with PBS twice, mitochondrial membrane potentials were monitored by determining the relative amounts of dual emission from a multiple fluorescence reader. Reactive oxygen species (ROS) were monitored with the oxidation sensitive fluorescent probe 2′,7′-dichlorodihydrofluorescein diacetate (DCF-DA).

### Quantitative real-time PCR (qRT-PCR)

Total mRNA of cells were extracted with TRIZOL reagent. First strand of cDNA was generated from 2 μg total RNA using oligo-dT primer and Superscript II Reverse Transcriptase (GIBCO BRL, Grand Island, NY, USA). qRT-PCR was run on an iCycler (Bio-rad, Hercules, USA) using validated primers and SYBR Premix Ex Taq II (Takara, Japan) for detection. The cycle number when the fluorescence first reached a preset threshold (Ct) was used to quantify the initial concentration of individual templates for expression of mRNA of genes of interest. Transcripts of the housekeeping gene GAPDH in the same incubations were used for internal normalization. Primer pairs were as follows: SOD1, forward 5′-GCA ATG TGA CTG CTG ACA AAG AT-3′ and reverse 5′-ATT ACA CCA CAA GCC AAA CGA CT-3′; SOD2, forward 5′-TGG ACA AAC CTC AGC CCT AAC-3′ and reverse 5′-GAA ACC AAG CCA ACC CCA AC-3′; GXP1, forward 5′-TGG ACC TTC ACC AGA CCT AC-3′ and reverse 5′-GGT TGA GGA GAG AAA CAC CA-3′; Catalase, forward 5′-TTC GGT TCT CCA CTG TTG CT-3′ and reverse 5′-GAT GTG TCT GAG GAT TTC TCT TTT G-3′; Nox1, forward 5′-TTT GGT TAG GGC TGA ATG T-3′ and reverse 5′-TTG TGG AAG GTG AGG TTG-3′; Nox4, forward 5′-ATG GTG GTG GTG CTA TTC-3′ and reverse 5′-GGG AGG GTG GGT ATC TAA-3′.

### Western blot analysis

Western blot analysis was performed as previously described [58].

### Tumorigenesis assay

Nude mice were purchased from the Sun Yat-Sen University (Guangzhou, China) Animal Center and raised under pathogen-free conditions. All animal studies were conducted in accordance with institutional guidelines for the care and use of experimental animals. MDA-MB-231 cells (2 × 10^6^ per mouse) were injected subcutaneously into the fourth right mammary fat pad at the base of the nipple of nude mice with 50% Matrigel (BD bioscience, Bedford, MA). When the tumor was visible (14 days post injection of tumor cells), all mice were randomly divided into XCT-790 and control group (*n* = 8 for each group). Mice of XCT-790 group were treated with XCT-790 (2 mg per kg, body weight) by tail vein injection for every three days (Six times together). Control group was treated with an equal volume of vehicle. Tumor growth and body weight were monitored every three days. The tumor volume was calculated using the formula V = 1/2 × larger diameter × (smaller diameter)^2^. When tumor volume at the control group reached approximate 1000 mm^3^ (35 days post injection of tumor cells), the animals were sacrificed, and the tumors were removed and weighed for Western blot analysis.

### Statistical analysis

All values were reported as mean ± SD of three independent experiments unless otherwise specified. Data were analyzed by two-tailed unpaired Student's *t*-test between two groups. The statistical analyses were performed using SPSS 17.0 for Windows. A *p*-value of < 0.05 was considered to be statistically significant.

## SUPPLEMENTARY MATERIALS FIGURES



## References

[1] Giguere V, Yang N, Segui P, Evans RM (1988). Identification of a new class of steroid hormone receptors. Nature.

[2] Giguere V (2008). Transcriptional control of energy homeostasis by the estrogen-related receptors. Endocr Rev.

[3] Chang CY, Kazmin D, Jasper JS, Kunder R, Zuercher WJ, McDonnell DP (2011). The Metabolic Regulator ERR alpha, a Downstream Target of HER2/IGF-1R, as a Therapeutic Target in Breast Cancer. Cancer Cell.

[4] Suzuki T, Miki Y, Moriya T, Shimada N, Ishida T, Hirakawa H, Ohuchi N, Sasano H (2004). Estrogen-related receptor alpha in human breast carcinoma as a potent prognostic factor. Cancer Res.

[5] Deblois G, Hall JA, Perry MC, Laganiere J, Ghahremani M, Park M, Hallett M, Giguere V (2009). Genome-wide identification of direct target genes implicates estrogen-related receptor alpha as a determinant of breast cancer heterogeneity. Cancer Res.

[6] Dufour CR, Wilson BJ, Huss JM, Kelly DP, Alaynick WA, Downes M, Evans RM, Blanchette M, Giguere V (2007). Genome-wide orchestration of cardiac functions by the orphan nuclear receptors ERRalpha and gamma. Cell Metab.

[7] Bianco S, Lanvin O, Tribollet V, Macari C, North S, Vanacker JM (2009). Modulating estrogen receptor-related receptor-alpha activity inhibits cell proliferation. J Biol Chem.

[8] Ao A, Wang H, Kamarajugadda S, Lu JR (2008). Involvement of estrogen-related receptors in transcriptional response to hypoxia and growth of solid tumors. Proc Natl Acad Sci.

[9] Stein RA, Gaillard S, McDonnell DP (2009). Estrogen-related receptor alpha induces the expression of vascular endothelial growth factor in breast cancer cells. J Steroid Biochem Mol Biol.

[10] Deblois G, Giguere V (2013). Oestrogen-related receptors in breast cancer: control of cellular metabolism and beyond. Nat Rev Cancer.

[11] Chang CY, McDonnell DP (2012). Molecular pathways: the metabolic regulator estrogen-related receptor alpha as a therapeutic target in cancer. Clin Cancer Res.

[12] Perou CM (2012). Molecular stratification of triple-negative breast cancers. Oncologist.

[13] Dent R, Trudeau M, Pritchard KI, Hanna WM, Kahn HK, Sawka CA, Lickley LA, Rawlinson E, Sun P, Narod SA (2007). Triple-negative breast cancer: clinical features and patterns of recurrence. Clin Cancer Res.

[14] Amir E, Ocana A, Freedman O, Clemons M, Seruga B (2010). Chemotherapy: dose-dense treatment for triple-negative breast cancer. Nat Rev Clin Oncol.

[15] Carey L, Winer E, Viale G, Cameron D, Gianni L (2010). Triple-negative breast cancer: disease entity or title of convenience?. Nat Rev Clin Oncol.

[16] Liedtke C, Mazouni C, Hess KR, Andre F, Tordai A, Mejia JA, Symmans WF, Gonzalez-Angulo AM, Hennessy B, Green M, Cristofanilli M, Hortobagyi GN, Pusztai L (2008). Response to neoadjuvant therapy and long-term survival in patients with triple-negative breast cancer. J Clin Oncol.

[17] Lehmann BD, Bauer JA, Chen X, Sanders ME, Chakravarthy AB, Shyr Y, Pietenpol JA (2011). Identification of human triple-negative breast cancer subtypes and preclinical models for selection of targeted therapies. J Clin Invest.

[18] Kirkpatrick P (2009). Targeting triple-negative breast cancer. Nat Rev Drug Discovery.

[19] Chisamore MJ, Wilkinson HA, Flores O, Chen JD (2009). Estrogen-related receptor-alpha antagonist inhibits both estrogen receptor-positive and estrogen receptor-negative breast tumor growth in mouse xenografts. Mol Cancer Ther.

[20] Wu YM, Chen ZJ, Liu H, Wei WD, Lu LL, Yang XL, Liang WT, Liu T, Liu HL, Du J, Wang HS (2015). Inhibition of ERR alpha suppresses epithelial mesenchymal transition of triple negative breast cancer cells by directly targeting fibronectin. Oncotarget.

[21] Manna S, Bostner J, Sun Y, Miller LD, Alayev A, Schwarts NS, Lager E, Fornander T, Nordenskjold B, Yu J, Stal O, Holz MK (2015). ERRalpha is a marker of tamoxifen response and survival in triple-negative breast cancer. Clin Cancer Res.

[22] Willy PJ, Murray IR, Qian J, Busch BB, Stevens WC, Martin R, Mohan R, Zhou S, Ordentlich P, Wei P, Sapp DW, Horlick RA, Heyman RA (2004). Regulation of PPARgamma coactivator 1alpha (PGC-1alpha) signaling by an estrogen-related receptor alpha (ERRalpha) ligand. Proc Natl Acad Sci.

[23] Busch BB, Stevens WC, Martin R, Ordentlich P, Zhou S, Sapp DW, Horlick RA, Mohan R (2004). Identification of a selective inverse agonist for the orphan nuclear receptor estrogen-related receptor alpha. J Med Chem.

[24] Raj L, Ide T, Gurkar AU, Foley M, Schenone M, Li X, Tolliday NJ, Golub TR, Carr SA, Shamji AF, Stern AM, Mandinova A, Schreiber SL (2011). Selective killing of cancer cells by a small molecule targeting the stress response to ROS. Nature.

[25] Ohkouchi S, Block GJ, Katsha AM, Kanehira M, Ebina M, Kikuchi T, Saijo Y, Nukiwa T, Prockop DJ (2012). Mesenchymal stromal cells protect cancer cells from ROS-induced apoptosis and enhance the Warburg effect by secreting STC1. Mol Ther.

[26] Nogueira V, Hay N (2013). Molecular pathways: reactive oxygen species homeostasis in cancer cells and implications for cancer therapy. Clin Cancer Res.

[27] Wu WS (2006). The signaling mechanism of ROS in tumor progression. Cancer Metastasis Rev.

[28] Verfaillie T, Rubio N, Garg AD, Bultynck G, Rizzuto R, Decuypere JP, Piette J, Linehan C, Gupta S, Samali A, Agostinis P (2012). PERK is required at the ER-mitochondrial contact sites to convey apoptosis after ROS-based ER stress. Cell Death Differ.

[29] Jones RG, Plas DR, Kubek S, Buzzai M, Mu J, Xu Y, Birnbaum MJ, Thompson CB (2005). AMP-activated protein kinase induces a p53-dependent metabolic checkpoint. Mol Cell.

[30] De Luca A, Maiello MR, D'Alessio A, Pergameno M, Normanno N (2012). The RAS/RAF/MEK/ERK and the PI3K/AKT signalling pathways: role in cancer pathogenesis and implications for therapeutic approaches. Expert Opin Ther Targets.

[31] Baud V, Karin M (2009). Is NF-kappaB a good target for cancer therapy? Hopes and pitfalls. Nat Rev Drug Discov.

[32] Deblois G, Chahrour G, Perry MC, Sylvain-Drolet G, Muller WJ, Giguere V (2010). Transcriptional control of the ERBB2 amplicon by ERRalpha and PGC-1beta promotes mammary gland tumorigenesis. Cancer Res.

[33] Bernatchez G, Giroux V, Lassalle T, Carpentier AC, Rivard N, Carrier JC (2013). ERRalpha metabolic nuclear receptor controls growth of colon cancer cells. Carcinogenesis.

[34] Stein RA, Chang CY, Kazmin DA, Way J, Schroeder T, Wergin M, Dewhirst MW, McDonnell DP (2008). Estrogen-related receptor alpha is critical for the growth of estrogen receptor-negative breast cancer. Cancer Res.

[35] Lam SS, Mak AS, Yam JW, Cheung AN, Ngan HY, Wong AS (2014). Targeting estrogen-related receptor alpha inhibits epithelial-to-mesenchymal transition and stem cell properties of ovarian cancer cells. Mol Ther.

[36] Sailland J, Tribollet V, Forcet C, Billon C, Barenton B, Carnesecchi J, Bachmann A, Gauthier KC, Yu S, Giguere V, Chan FL, Vanacker JM (2014). Estrogen-related receptor a decreases RHOA stability to induce orientated cell migration. Proc Natl Acad Sci.

[37] Dwyer MA, Joseph JD, Wade HE, Eaton ML, Kunder RS, Kazmin D, Chang CY, McDonnell DP (2010). WNT11 Expression Is Induced by Estrogen-Related Receptor alpha and beta-Catenin and Acts in an Autocrine Manner to Increase Cancer Cell Migration. Cancer Res.

[38] Rangwala SM, Li XY, Lindsley L, Wang XM, Shaughnessy S, Daniels TG, Szustakowski J, Nirmala NR, Wu ZD, Stevenson SC (2007). Estrogen-related receptor alpha is essential for the expression of antioxidant protection genes and mitochondrial function. Biochem Biophy Res Com.

[39] Wang J, Wang Y, Wong C (2010). Oestrogen-related receptor alpha inverse agonist XCT-790 arrests A549 lung cancer cell population growth by inducing mitochondrial reactive oxygen species production. Cell Prolif.

[40] Trachootham D, Alexandre J, Huang P (2009). Targeting cancer cells by ROS-mediated mechanisms: a radical therapeutic approach?. Nat Rev Drug Discov.

[41] Schumacker PT (2006). Reactive oxygen species in cancer cells: live by the sword, die by the sword. Cancer Cell.

[42] Boyce M, Yuan J (2006). Cellular response to endoplasmic reticulum stress: a matter of life or death. Cell Death Differ.

[43] Yoshida H, Matsui T, Yamamoto A, Okada T, Mori K (2001). XBP1 mRNA is induced by ATF6 and spliced by IRE1 in response to ER stress to produce a highly active transcription factor. Cell.

[44] Ishida Y, Nagata K (2009). Autophagy eliminates a specific species of misfolded procollagen and plays a protective role in cell survival against ER stress. Autophagy.

[45] Moon DO, Park SY, Choi YH, Ahn JS, Kim GY (2011). Guggulsterone sensitizes hepatoma cells to TRAIL-induced apoptosis through the induction of CHOP-dependent DR5: Involvement of ROS-dependent ER-stress. Biochem Pharmacol.

[46] Gao Z, Sarsour EH, Kalen AL, Li L, Kumar MG, Goswami PC (2008). Late ROS accumulation and radiosensitivity in SOD1-overexpressing human glioma cells. Free Radic Biol Med.

[47] Hamanaka RB, Chandel NS (2010). Mitochondrial reactive oxygen species regulate cellular signaling and dictate biological outcomes. Trends Biochem Sci.

[48] Hancock JT, Desikan R, Neill SJ (2001). Role of reactive oxygen species in cell signalling pathways. Biochem Soc Trans.

[49] Zhang W, Liu HT (2002). MAPK signal pathways in the regulation of cell proliferation in mammalian cells. Cell Res.

[50] Wei W, Chen ZJ, Zhang KS, Yang XL, Wu YM, Chen XH, Huang HB, Liu HL, Cai SH, Du J, Wang HS (2014). The activation of G protein-coupled receptor 30 (GPR30) inhibits proliferation of estrogen receptor-negative breast cancer cells *in vitro* and *in vivo*. Cell Death Dis.

[51] Baumer AT, Ten Freyhaus H, Sauer H, Wartenberg M, Kappert K, Schnabel P, Konkol C, Hescheler J, Vantler M, Rosenkranz S (2008). Phosphatidylinositol 3-kinase-dependent membrane recruitment of Rac-1 and p47phox is critical for alpha-platelet-derived growth factor receptor-induced production of reactive oxygen species. J Biol Chem.

[52] Dumont A, Hehner SP, Hofmann TG, Ueffing M, Droge W, Schmitz ML (1999). Hydrogen peroxide-induced apoptosis is CD95-independent, requires the release of mitochondria-derived reactive oxygen species and the activation of NF-kappaB. Oncogene.

[53] Bian X, McAllister-Lucas LM, Shao F, Schumacher KR, Feng Z, Porter AG, Castle VP, Opipari AW (2001). NF-kappa B activation mediates doxorubicin-induced cell death in N-type neuroblastoma cells. J Biol Chem.

[54] Ahn J, Won M, Choi JH, Kim YS, Jung CR, Im DS, Kyun ML, Lee K, Song KB, Chung KS (2013). Reactive oxygen species-mediated activation of the Akt/ASK1/p38 signaling cascade and p21(Cip1) downregulation are required for shikonin-induced apoptosis. Apoptosis.

[55] Dan HC, Cooper MJ, Cogswell PC, Duncan JA, Ting JP, Baldwin AS (2008). Akt-dependent regulation of NF-κB is controlled by mTOR and Raptor in association with IKK. Genes Dev.

[56] Ge LC, Chen ZJ, Liu HY, Zhang KS, Liu H, Huang HB, Zhang G, Wong CKC, Giesy JP, Du J, Wang HS (2014). Involvement of activating ERK1/2 through G protein coupled receptor 30 and estrogen receptor alpha/beta in low doses of bisphenol A promoting growth of Sertoli TM4 cells. Toxicol Lett.

[57] Zhang YH, Wang Y, Yusufali AH, Ashby F, Zhang D, Yin ZF, Aslanidi GV, Srivastava A, Ling CQ, Ling C (2014). Cytotoxic genes from traditional Chinese medicine inhibit tumor growth both *in vitro* and *in vivo*. J Integr Med.

[58] Jiang GM, Wang HS, Zhang F, Zhang KS, Liu ZC, Fang R, Wang H, Cai SH, Du J (2013). Histone deacetylase inhibitor induction of epithelial-mesenchymal transitions via up-regulation of Snail facilitates cancer progression. BBA-Mol Cell Res.

