# Supplementary material for: Inverse agonist of estrogen-related receptor α suppresses the growth of triple negative breast cancer cells through ROS generation and interaction with multiple cell signaling pathways
Source: Oncotarget. 2016 Feb 9;7(11):12568–81. doi: 10.18632/oncotarget.7276 (PMC4914305; doi:10.18632/oncotarget.7276)
Supplement: Supplementary file 1 [file oncotarget-07-12568-s001.pdf]

Inverse agonist of estrogen-related receptor α suppresses the growth of triple negative breast cancer cells through ROS generation and interaction with multiple cell signaling pathways

Supplementary Materials

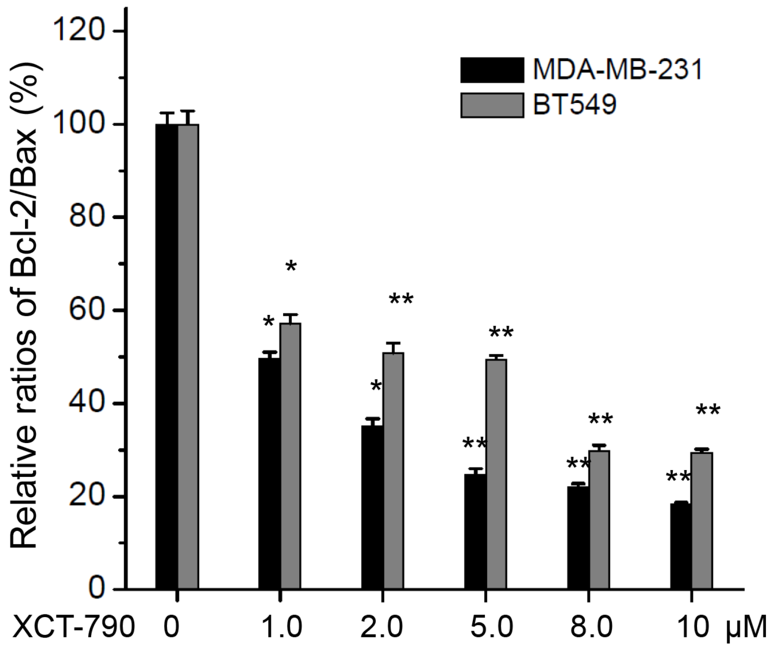

Supplementary Figure S1: The relative ratios of Bcl-2 to Bax in TNBC cells treated with XCT-790. MDA-MB-231 and BT549 cells treated with XCT-790 as the indicated concentrations for 48 h, and then Bcl-2 and Bax protein expression levels were analyzed by Western-blot analysis. Then ratios of Bcl-2/Bax were quantified. Data were presented as means ± SD of three independent experiments. **p* < 0.05 compared with control, ***p* < 0.01 compared with control.

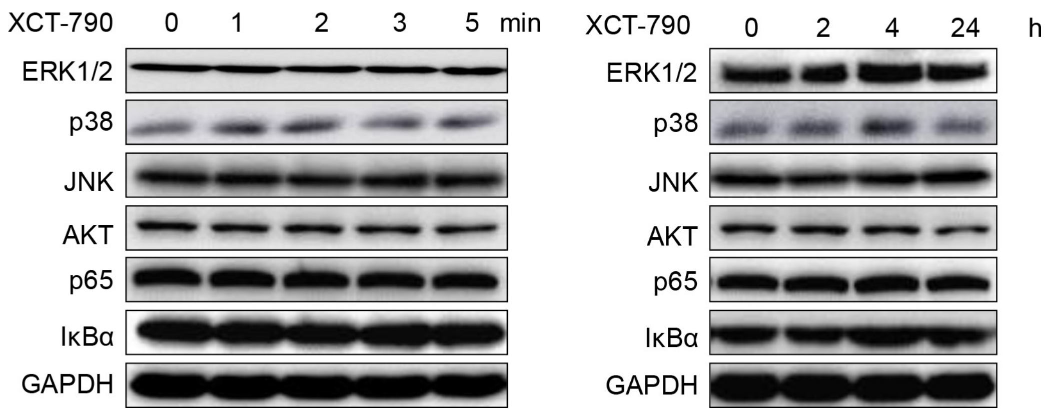

Supplementary Figure S2: The effects of XCT-790 on expression of MAPK, PI3K/Akt, NF-κB signals. MDA-MB-231 cells were treated with 5 μM XCT-790 for the indicated times, and then the total expression of these signal molecules were measured by Western blot analysis.

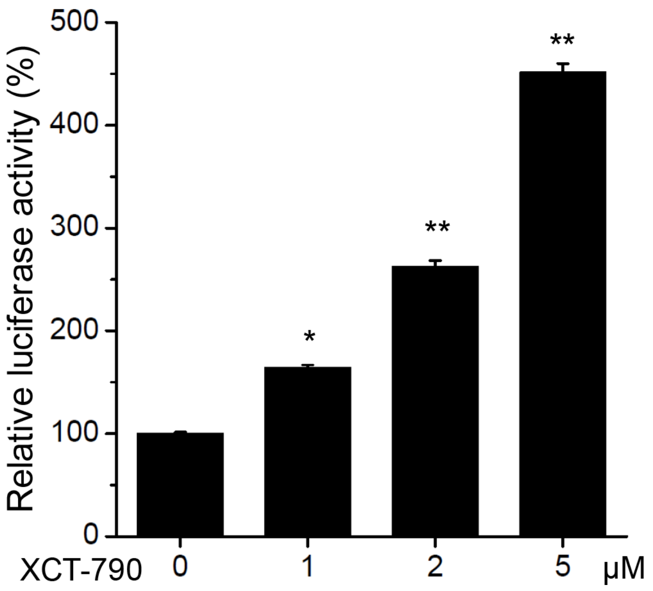

Supplementary Figure S3: XCT-790 increases the transcription activities of NF-κB. MDA-MB-231 cells were transfected with a luciferase reporter construct containing 5 copies of the κB site plasmid for 24 h and then treated with increasing concentrations of XCT-790 for 6 h, then the lysates were assayed. Shown are relative luciferase activities normalized to Renilla activities. Data represent the average of three independent experiments. **p* < 0.05 compared with control, ***p* < 0.01 compared with control.

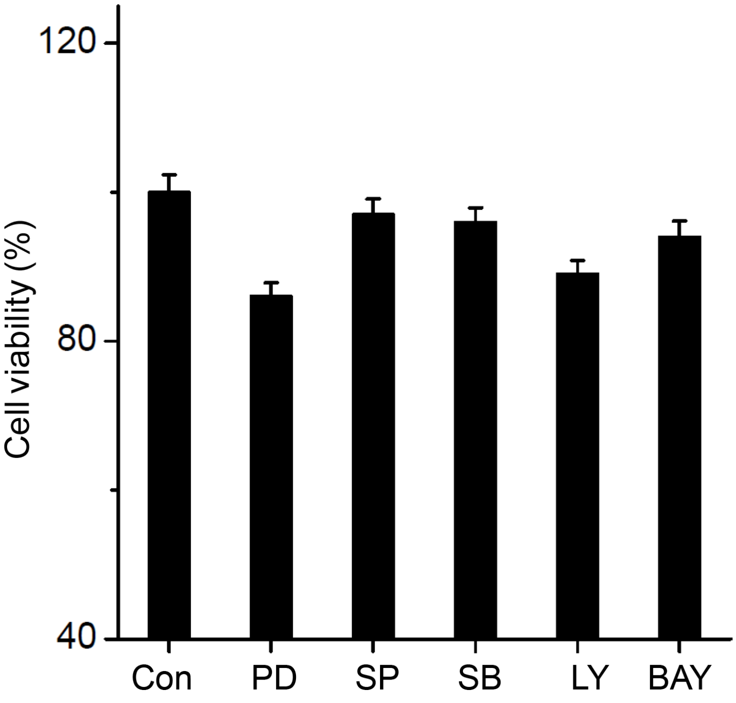

Supplementary Figure S4: The effects of inhibitors on the proliferation of MDA-MB-231 cells. MDA-MB-231 cells were pretreated with 10 μM ERK1/2 inhibitor PD98059(PD), JNK inhibitor SP600125 (SP), p38-MAPK inhibitor SB203580 (SB), PI3K inhibitor LY294002 (LY), or NF-κB inhibitor BAY11-7082 (BAY) for 48 h, the cell viability was measured by CCK-8 assay. Data were presented as means ± SD of three independent experiments.
